# Serial block face scanning electron microscopy reveals region-dependent remodelling of transverse tubules post-myocardial infarction

**DOI:** 10.1098/rstb.2021.0331

**Published:** 2022-11-21

**Authors:** Tharushi Perera, Charlene Pius, Barbara Niort, Emma J. Radcliffe, Katharine M. Dibb, Andrew W. Trafford, Christian Pinali

**Affiliations:** Unit of Cardiac Physiology, Division of Cardiovascular Sciences, Faculty of Biology Medicine and Health, University of Manchester, Manchester Academic Health Sciences Centre, 46 Grafton Street, Manchester M13 9NT, UK

**Keywords:** transverse tubules, cardiomyocyte, myocardial infarction, remodelling, three-dimensional electron microscopy

## Abstract

The highly organized transverse tubule (t-tubule) network facilitates cardiac excitation–contraction coupling and synchronous cardiac myocyte contraction. In cardiac failure secondary to myocardial infarction (MI), changes in the structure and organization of t-tubules result in impaired cardiac contractility. However, there is still little knowledge on the regional variation of t-tubule remodelling in cardiac failure post-MI. Here, we investigate post-MI t-tubule remodelling in infarct border and remote regions, using serial block face scanning electron microscopy (SBF-SEM) applied to a translationally relevant sheep ischaemia reperfusion MI model and matched controls. We performed minimally invasive coronary angioplasty of the left anterior descending artery, followed by reperfusion after 90 min to establish the MI model. Left ventricular tissues obtained from control and MI hearts eight weeks post-MI were imaged using SBF-SEM. Image analysis generated three-dimensional reconstructions of the t-tubular network in control, MI border and remote regions. Quantitative analysis revealed that the MI border region was characterized by t-tubule depletion and fragmentation, dilation of surviving t-tubules and t-tubule elongation. This study highlights region-dependent remodelling of the tubular network post-MI and may provide novel localized therapeutic targets aimed at preservation or restoration of the t-tubules to manage cardiac contractility post-MI.

This article is part of the theme issue ‘The cardiomyocyte: new revelations on the interplay between architecture and function in growth, health, and disease’.

## 1. Introduction

During the cardiac action potential, EC coupling is initiated by opening of the sarcolemmal L-type calcium channels (LTCCs), leading to an influx of Ca^2+^, triggering a larger Ca^2+^ release from the intracellular Ca^2+^ store, the sarcoplasmic reticulum (SR), via the ryanodine receptors (RyRs). Following myofilament contraction, relaxation occurs by means of Ca^2+^ re-uptake via the SR Ca^2+^ ATPase (SERCA) and Ca^2+^ efflux from the cell via the sodium–calcium exchanger, completing the cycle with the relaxation of the contractile apparatus [1].

In healthy mammalian ventricular myocytes, transverse tubules (t-tubules) are pipe-like invaginations of the sarcolemma where LTCCs are concentrated and closely opposed to RyRs, which are distributed throughout the cytosol on the SR membrane and together form a functional unit termed the cardiac dyad [2]. In healthy cells, t-tubules are found at the *z*-lines and orientated perpendicular to the sarcolemma, demonstrating a centripetal spoke-like arrangement when cells are observed in cross-section [3]. In addition to defining transverse elements, the tubular network contains some longitudinal elements branching from the t-tubules and extending between *z*-lines [4].

In various cardiovascular diseases, remodelling of the ultrastructure of cardiac myocytes is noted, especially involving t-tubules [3,5,6]. For example, initial ultrastructural two-dimensional (2D) electron microscopy (EM) work [7] noted an increase in t-tubule membrane area in hypertrophied rat hearts. Subsequent 2D EM studies from several groups [8–10] observed dilation or loss of t-tubules in ischaemic and dilated cardiomyopathies. Further imaging studies using scanning ion conductance microscopy and confocal microscopy identified marked disorganization and loss of t-tubules in failing hearts in a range of conditions and experimental systems, including congestive heart failure [2], dilated cardiomyopathy [11], chronic myocardial infarction (MI) [11] and heart failure secondary to thoracic banding [12]. However, these models, albeit fundamental to further our understanding, are of limited translational relevance to cardiac failure post-myocardial insult in humans.

In fact, using two-photon laser scanning microscopy, Ohler *et al.* [13] found no changes in the organization and structure of t-tubules in failing human hearts, which may be accounted for by selection of relatively healthy cardiac myocytes during cell isolation. By contrast Seidel *et al.* [14] demonstrated sheet-like remodelling of the t-tubule network in failing human hearts. These differences may also be reconciled by tissue heterogeneity, whereby Crossman *et al.* [3] found that a diseased t-tubule area can coexist with an adjacent area consisting of healthy t-tubules within a human failing myocyte. As well as t-tubule loss and disorganization, more recent work [15,16] has reported a striking increase in longitudinal tubules combined with a decrease in transverse tubules within the tubular network in cardiac failure secondary to MI and Serca2 gene deletion.

As a result of t-tubule remodelling, the regulation of intracellular calcium and EC coupling will be altered in heart failure. For example, delayed Ca^2+^ release and reduced CICR were associated with a reduction of t-tubules in heart failure [2,17]. In addition, uncoupling of the cardiac dyad and subsequent orphaning of RyRs resulted in dyssynchronous Ca^2+^ release [18,19] and increased spontaneous arrhythmogenic Ca^2+^ sparks [20]. Thus, structural alterations to t-tubules have been associated with the hallmark sequelae of contractile dysfunction and arrhythmias in failing hearts.

Importantly, there is still uncertainty regarding whether disorganization and loss of t-tubules occur uniformly across different regions of the heart. To this end, the present study aims to investigate regional remodelling of t-tubules using a translationally relevant sheep ischaemia reperfusion (IR) MI model, eight weeks after initiation of the ischaemic episode. The IR model is translationally relevant, as it recapitulates the pathophysiological processes that underpin the initial ischaemic insult due to interruption of blood flow, and subsequent injury by reperfusion, which occurs in human MI following coronary intervention procedures [21–23]. Moreover, the use of sheep enhances the translational value of this model as the sheep heart shares similar coronary anatomy [24] and cardiac function to humans [25], in comparison with other animal species. Additionally, the lack of a collateral coronary network in sheep increases their suitability for myocardial ischaemia research [26]. We have used serial block face scanning electron microscopy (SBF-SEM) to study the ultrastructural changes occurring in regions of the heart proximal (border region) and distal (remote region) to the infarct in a translationally relevant MI model. The remodelling of the tubular network under the conditions studied here appeared to be influenced by the distance to the infarct focus, with depletion, fragmentation and elongation of t-tubules in the border region. Ultimately, identification of the altered ultrastructure may potentially enable the development of new therapies that are region-based in the heart to target specific ultrastructural changes in the management of contractile dysfunction and arrhythmias in MI patients.

## 2. Material and methods

### (a) Sheep ischaemia reperfusion myocardial infarction model

All experimental procedures were performed in accordance with the United Kingdom (UK) Animals (Scientific Procedures) Act 1986 and European Union Directive 2010/63, with local ethical approval obtained from the University of Manchester (Manchester, UK) Animal Welfare and Ethical Review Board. Six adult (approx. 18 months) female Welsh Mountain sheep were used in this study (*N* = 3 MI, *N* = 3 control).

Following overnight fasting, anaesthesia was induced using 5% isoflurane (Santa Cruz Biotechnology, USA) in a 50 : 50 v/v mix of nitrous oxide and oxygen (O_2_). After intubation, anaesthesia was maintained using 1–3% isoflurane in O_2_ with mechanical ventilation throughout the procedure. Arterial blood pressure, heart rate, O_2_ saturation and cardiac activity were closely monitored during the procedure. Fluids and drugs administered during the surgery included: intravenous (IV) maintenance fluid (0.9% NaCl, Baxter USA) to compensate for blood loss, and injections of meloxicam (0.5 mg kg^−1^ Metacam^®^, Boehringer Ingelheim, Germany) and amoxicillin (20 µg kg^−1^ Betamox^®^, Norbrook, UK) as prophylactic analgesic and antibiotic treatments.

### (b) Myocardial infarction induction

Under fluoroscopic guidance, the left coronary ostia was engaged with a 6 Fr guide catheter, a 0.014 inch guide wire was advanced along the left anterior descending (LAD) artery, and an angioplasty balloon was positioned distal to the second diagonal artery. To simulate the occurrence of an MI episode in humans, balloon occlusion was in place for 90 min. The angioplasty balloon was then deflated, all equipment was removed and perfusion was restored, with reperfusion confirmed by fluoroscopic contrast imaging. Following the procedure, animals were allowed to recover and monitored for a period of eight weeks. Animal weight, wellbeing and wound healing were closely monitored to ensure adequate recovery. *In vivo* examinations were carried out at one, three and eight weeks post-surgery, involving cardiac troponin blood analysis, electrocardiography and echocardiography to establish cardiac failure via evaluation of left ventricular ejection fraction (LVEF). Eight weeks after MI, animals (or un-operated age-matched control animals) were humanely killed using IV pentobarbitone (200 mg kg^−1^ Pentoject, Animalcare, UK); 10 000 IU heparin was administered intravenously to prevent coagulation.

### (c) Sample preparation

After euthanasia, sheep hearts were extracted and perfused in calcium-free Tyrode's solution. Left ventricular tissue cubes (approx. 1 mm^3^) were excised from the myocardium of control hearts, and from border (less than 0.5 cm adjacent to the scar) and remote (defined as being at least 1.5 cm from the infarct and showing no epicardial evidence of scar formation) regions of infarcted hearts. Samples were immersion-fixed in Karnovsky's fixative. Following washings in sodium cacodylate buffer, samples were subsequently stained using 2% osmium tetroxide and 1.5% potassium ferrocyanide; 1% tannic acid; 2% osmium tetroxide and 1% uranyl acetate, with washings in water following each staining step. After staining, samples were dehydrated in ethanol ascending series (50, 70, 90, 100 and 100%), followed by further dehydration in pure acetone. Infiltration was carried out using increasing concentrations of TAAB 812 hard resin (25, 50, 75 and 100%) mixed with acetone. Finally, the samples were embedded in pure resin and cured at 60°C for 36 h.

### (d) Three-dimensional image acquisition and image analysis

Samples were trimmed into approximately 0.5 mm^3^ portions out of the plastic blocks, glued onto cryo pins sputter-coated in gold–palladium and placed into the Gatan 3View device, inside a Quanta 250 FEG scanning electron microscope (SEM) operated at 3.5 kV and 0.44 Torr. Serial sections were cut at 50 nm and subsequent images were taken, generating a stack of images acquired in the Gatan ‘dm4’ format, with a nominal voxel size of 8–11 nm px^−1^ (*X*, *Y*) by 50 nm px^−1^ (*Z*).

The resulting stacks of images were visualized using Fiji [27] or 3dmod [28], with image segmentation and volumetric analysis performed in 3dmod. For three-dimensional (3D) reconstructions, three cells from the proximal and distal regions from each MI animal and controls were randomly chosen. For each cell, a portion projected along 100 images was selected. t-Tubules in this portion were manually segmented on every image, and their volume, surface area, diameter and length were measured using 3dmod.

t-Tubule lengths were measured manually using the length of the longest branch. For a given t-tubule, *equivalent diameters* were derived from the t-tubule contours based on the approximation that they are circles, using the equation:

$$\mathrm{equivalent}\;\mathrm{diameter}=\frac{\sum_{i=1}^{n}({\mathrm{contour}}_{i})}{n\pi },$$

where contour*_i_* is a contour of the t-tubule and *n* is the total number of contours for the same t-tubule. For each cell, length and diameter were calculated for 15 randomly chosen t-tubules (using the RAND function in Microsoft Excel 2016). A complete list of t-tubule measurements can be found in supplementary material.

### (e) Statistical analysis

Statistical analysis was performed using SAS OnDemand for Academics. Normality of data (proc UNIVARIATE) was confirmed using a Shapiro–Wilk or Kolmogorov–Smirnov test. Mean value ± s.e. was calculated for all normally distributed data (proc MEANS). Log_10_ or square root transforms were used where data were not normally distributed. As we have described previously [19], owing to the clustered (hierarchical) nature of the experimental data (multiple observations from each cell and animal), linear mixed model (proc MIXED) analysis with maximum-likelihood estimation was used to determine the statistical significance between groups. Normality of residual distribution was used to confirm appropriateness of the linear model. To compare LVEF at baseline and eight weeks post-MI a paired *t*-test was used. Data were considered significant at *p* = 0.05 and actual *p*-values are presented in the figures.

## 3. Results

### (a) Effectiveness of the sheep ischaemia reperfusion myocardial infarction model

**Figure 1.**
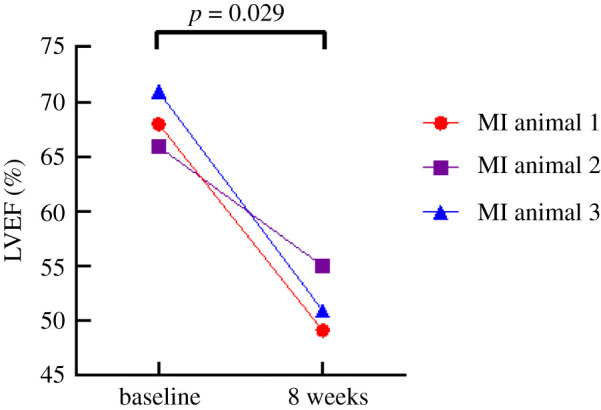
Reduction in LVEF from baseline to eight weeks post-MI. Mean LVEF eight weeks post-MI (52 ± 2%) is reduced by 16 ± 4% when compared with baseline mean LVEF (68 ± 2%). Paired *t-*test, *p* = 0.029, GraphPad Prism 8.4.3.

*In vivo* contractility was evaluated using transthoracic long-axis view LVEF measurements in experimental sheep model (*N* = 3), collected at baseline prior to the MI surgery and at eight weeks following MI. As presented in figure 1, echocardiography data demonstrated that LVEF, on average, was reduced by 16 ± 4% at eight weeks post-MI (baseline: 68 ± 2%; MI: 52 ± 2%; *p* = 0.029), confirming hypokinesis and mild cardiac dysfunction in these animals.

### (b) Morphology of ventricular t-tubules in healthy sheep

#### (i) Arrangement of the tubular network

**Figure 2.**
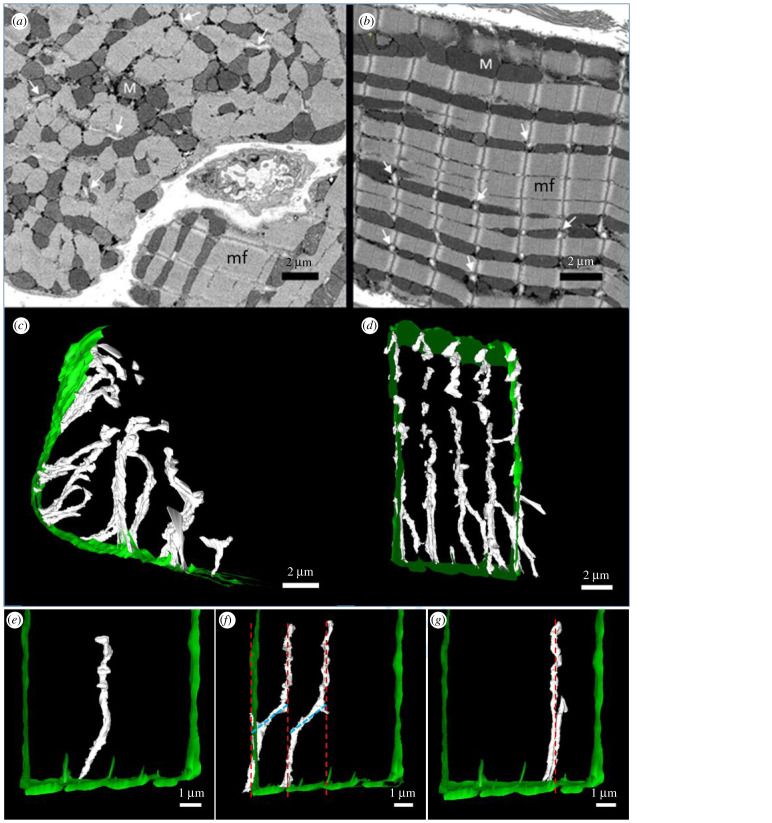
SBF-SEM images and 3D reconstructions of the t-tubular network in healthy sheep cardiac myocytes. (*a*,*b*), SBF-SEM images illustrating key features in control cardiac myocytes, cut in transverse (*a*) and longitudinal (*b*) sections. t-Tubules are indicated by white arrows. M: mitochondria; mf: myofilaments. (*c*) 3D reconstruction of the t-tubular network in a control cardiac myocyte in transverse view, where t-tubules (white) adopt a radial orientation extending from the sarcolemma (green) to the cell centre. (*d*) 3D reconstruction of the t-tubular network in a healthy cardiac myocyte in longitudinal view, displaying an ordered arrangement of t-tubules (white) arranged perpendicular to the sarcolemma (horizontal green regions at the top and bottom) and regularly along *z*-lines. Vertical green lines are present to delimit the region. (*e*) Focus on a single t-tubule showing dilations and narrowings along its length. (*f*) Focus on a pair of t-tubules showing a longitudinal arrangement (blue dashed lines) at the crossover between *z*-lines (red dashed lines). (*g*) Focus on twin tubules projecting from the same region on either sides of the *z*-line (red dashed line). (*a*–*d*) scale bars: 2 µm; (*e*–*g*) scale bars: 1 µm.

SBF-SEM images of healthy sheep cardiac myocytes are shown in figure 2, in transverse (figure 2*a*) and longitudinal (figure 2*b*) views. The images show bands of mitochondria (dark grey, M) located between myofibrils (light grey, mf). By contrast, t-tubules appeared very light grey, enabling effective segmentation of the tubular network. 3D rendering showed that t-tubules formed a highly organized network arranged regularly along *z*-lines and perpendicular to the sarcolemma (figure 2*c,d*). As illustrated by figure 2*c*, t-tubules displayed a centripetal spoke-like organization in transverse section, originating at the sarcolemma and extending towards the cell centre.

#### (ii) t-Tubule morphology

**Figure 3.**
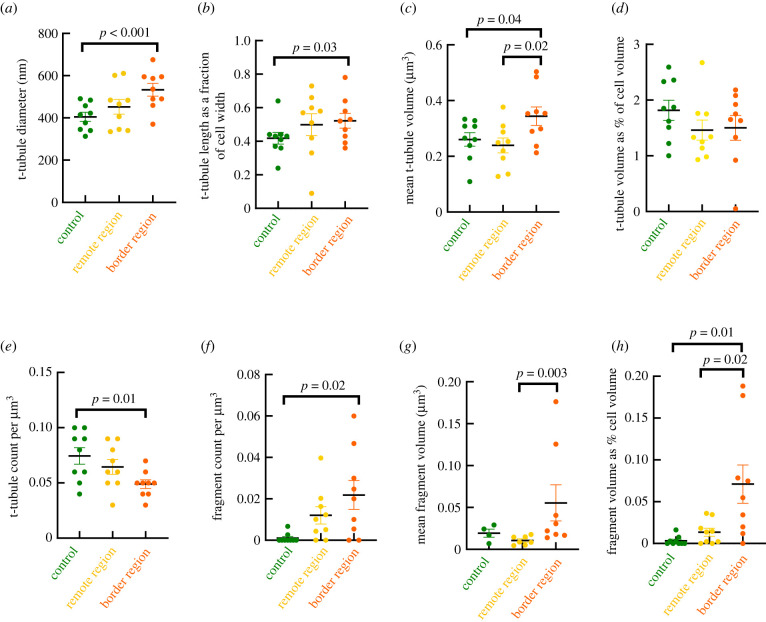
Comparison of t-tubule morphology between control, MI remote and border groups. (*a*–*h*) Comparison of t-tubule morphological features between control (green), MI remote (yellow) and border (orange) regions, illustrating: (*a*) increment of t-tubule diameter in border region compared with control; (*b*) increment of t-tubule length as a fraction of the cell in border region compared with control; (*c*) changes to the t-tubule volume in the border region compared with control and remote region; (*d*) volume occupied by t-tubules in the three different regions as a fraction of the cell volume; (*e*) the depletion of t-tubules in border region compared with control; (*f*) increment of fragments in border region compared with control; (*g*) increment in mean fragment volume in border region compared with remote region; (*h*) increment of fragment volume as a fraction of cell volume in border region compared with control and remote region. *p*-values demonstrating significant differences (*p* < 0.05) between groups are indicated.

Figure 2*e* shows a ‘typical’ t-tubule in a control cardiac myocyte, which displays a thin and long column-like structure with some narrowings and dilations along its length. t-Tubules from control hearts had an average diameter of 405 ± 22 nm (figure 3*a*; 135 randomly selected tubules from *n* = 9 cells and *N* = 3 sheep). To account for any influence of changes in cell width in the observed parameters, t-tubule lengths were normalized to the cell width to better understand how much they penetrate inside the cell. Using nanoscale analysis, we report for the first time to our knowledge, that the average t-tubule length as a fraction of cell width is 0.41 ± 0.04 in healthy sheep cardiac myocytes, which indicates that the majority of t-tubules penetrated slightly less than half of the cell width (figure 3*b*). High-resolution 3D reconstructions (308 t-tubules; *n* = 9 cells from *N =* 3 sheep) showed that the average volume per t-tubule is 0.25 ± 0.014 µm^3^, occupying 1.8 ± 0.2% of the total cell volume (figure 3*c*,*d*), with a mean t-tubular surface area of 5.7 ± 0.3 µm^2^. In addition, t-tubule density expressed as t-tubule count per unit cell volume was 0.07 ± 0.007 t-tubules per μm^3^ in healthy sheep cardiac myocytes (figure 3*e*). Moreover, it was evident that some t-tubules contained longitudinal components, which run perpendicular to the t-tubules at the crossover to adjacent *z*-lines without joining neighbouring t-tubules, as presented in figure 2*f*. As a general rule, we expect to find only one t-tubule per *z*-line; however, on rare occasions t-tubules traversed the cell in pairs, henceforth referred to as ‘twin tubules’ (figure 2*g*). These twin tubules began from the same area of the sarcolemma, penetrated the cell positioned on either side of a *z*-line, eventually diverging from each other the further they entered the cell.

### (c) Regional changes in t-tubule remodelling following myocardial infarction

#### (i) t-Tubular disorganization and fragmentation in the infarct border region

**Figure 4.**
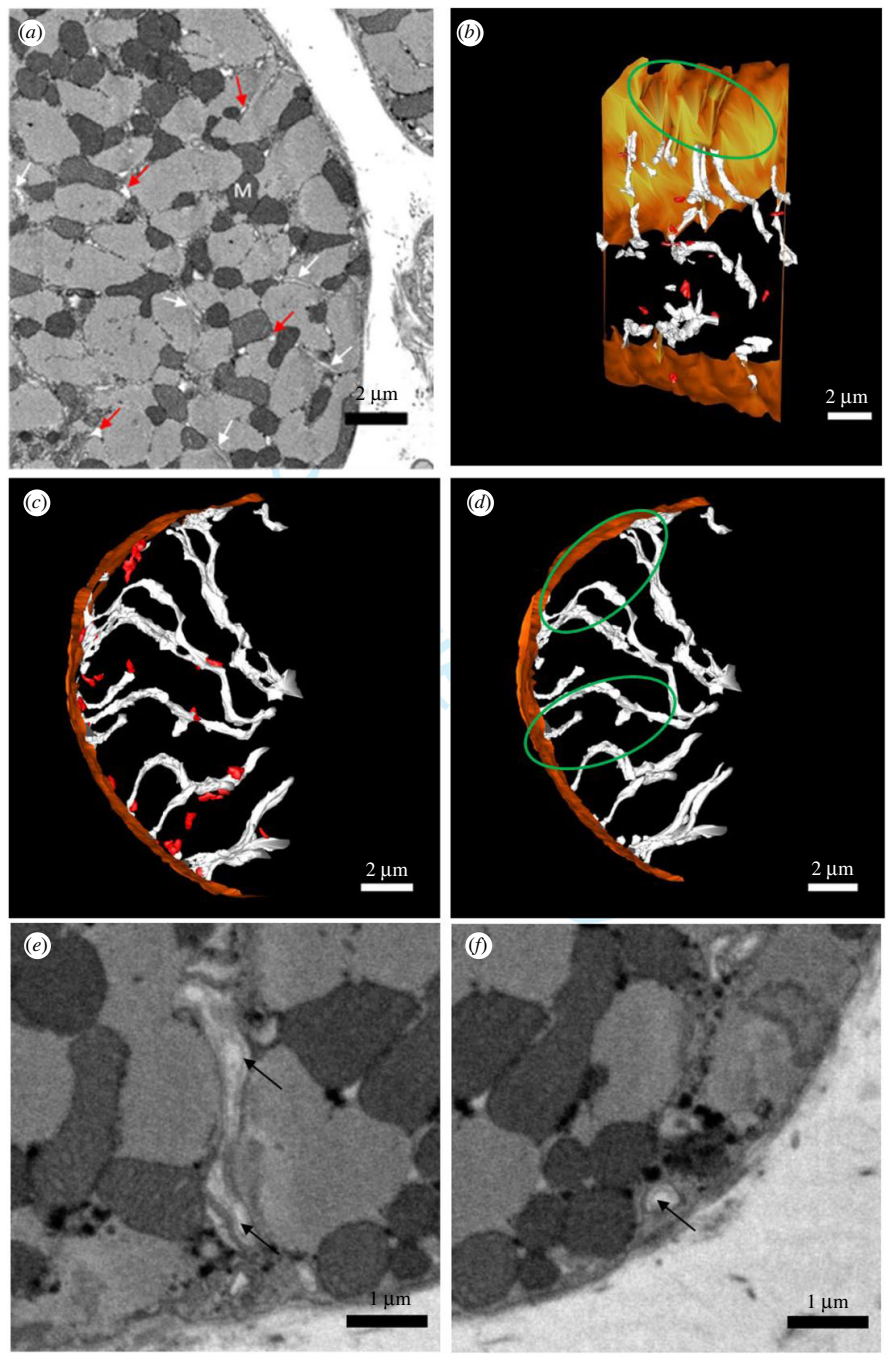
SBF-SEM images and 3D reconstructions of the t-tubular network in sheep border MI. (*a*) SBF-SEM image illustrating key features in a border region cell, in transverse section. t-Tubules are indicated by white arrows, and fragments by red arrows. M: mitochondria. (*b*) 3D reconstruction of the t-tubular network (white) in a border region cell in longitudinal view, displaying disorganization of t-tubules, t-tubule fragmentation (red) and regional t-tubule loss, indicated by a green ellipse. (*c*) 3D reconstruction of the t-tubular network (white) in a border region cell, in transverse view, illustrating extensive fragmentation (red). (*d*) 3D reconstruction of the border region cell shown in (*c*), with its fragments removed, displaying the underlying t-tubule network (white). Green ellipses indicate regions of t-tubule loss. (*e*) t-Tubules in light grey lined by the basement membrane indicated by black arrows. (*f*) Fragment in light grey preserving the basement membrane indicated by black arrow. (*a*–*d*) Scale bars: 2 µm; (*e*,*f*) scale bars: 1 µm.

An exemplar SBF-SEM image of cardiac myocytes from the infarct border region is presented in figure 4*a*, where seemingly there is no gross remodelling of the cardiac myocyte. However, 3D reconstruction revealed features difficult to detect with 2D image analysis. Once segmented, in comparison with control, the tubular network appeared highly disorganized, as t-tubules were not arranged regularly along *z*-lines in the MI border region, and t-tubules were also fragmented (figure 4*b*,*c*). Fragments are remnants of t-tubules not connected to the extracellular space and are characterized by an inner dark ring (figure 4*f*) of the basement membrane as often seen in t-tubules (figure 4*e*). This feature was used to distinguish between proper t-tubule fragments and vacuoles of other origin. Albeit very rarely found also in controls, t-tubule fragments predominated in cells from the MI border region, as shown in figure 4*b*,*c*. The number of fragments per μm^3^ was increased in the border region compared with control (border: 0.02 ± 0.007 fragment μm^−^^3^; control: 0.001 ± 0.001 fragment μm^−^^3^; *p* = 0.02; figure 3*f*), and the relative cell volume occupied by fragments was also greater in the border region compared with control (border: 0.071 ± 0.023%; control: 0.003 ± 0.002%; *p* = 0.01; figure 3*h*). Quantitative analysis of the mean fragment volume revealed a higher mean volume per fragment in the border region (0.06 ± 0.02 µm^3^, 89 fragments, *n* = 8 cells, *N* = 3 sheep), compared with remote (0.01 ± 0.002 µm^3^, 44 fragments, *n* = 7 cells, *N* = 3 sheep; *p* = 0.003), while there was no significant difference with control region (0.02 ± 0.005 µm^3^, 7 fragments, *n* = 4 cells, *N* = 3 sheep; *p* = 0.44), as shown in figure 3*g*.

#### (ii) Regional t-tubule loss and dilation of surviving t-tubules

Figure 4*d* illustrates a 3D reconstruction of the underlying tubular network in an MI border region cell where fragments have been removed. As visualized in figure 4*b,d*, it is apparent that there were some areas of t-tubule depletion (green ellipse), indicating intracellular heterogeneity of the tubular network secondary to post-MI remodelling. Quantitative analysis of the t-tubular count per cell volume confirmed these findings, showing a reduction in t-tubule density in the MI border region compared with control (border: 0.05 ± 0.004 tubule μm^−^^3^, *n* = 9 cells, *N* = 3 sheep; control: 0.07 ± 0.007 tubule μm^−^^3^, *n* = 9 cells, *N* = 3 sheep; *p* = 0.01; figure 3*e*), highlighting a significant loss of t-tubule density in the infarct border region following MI. In addition to t-tubule loss, we found that the remaining t-tubules in the MI border region were dilated; the mean t-tubule diameter was significantly increased in the border region compared with healthy cardiac myocytes (border: 533 ± 30 nm, 132 tubules, *n* = 9 cells, *N* = 3 sheep; control: 405 ± 22 nm, 135 tubules, *n* = 9 cells, *N* = 3 sheep; *p* < 0.001; figure 3*a*). In line with increased t-tubule diameter, mean t-tubule volume was increased compared with control (border: 0.37 ± 0.024 µm^3^, 227 tubules, *n* = 9 cells, *N* = 3 sheep; control: 0.25 ± 0.014 µm^3^, 308 tubules, *n* = 9 cells, *N* = 3 sheep; *p* = 0.04; figure 3*c*); similarly, surface area trended toward an increase in the border region compared with control (border: 6.8 ± 0.4 µm^2^, 227 tubules, *n* = 9 cells, *N* = 3 sheep; control: 5.7 ± 0.3 µm^2^, 308 tubules, *n* = 9 cells, *N* = 3 sheep; *p* = 0.15), supporting further the t-tubule remodelling in the border region following MI.

#### (iii) Increased t-tubule length in the border region

t-Tubule length as a fraction of the cell width was increased in the border region, compared with healthy cardiac myocytes (border: 0.52 ± 0.03, 132 tubules, *n = 9* cells*, N* = 3 sheep; control: 0.41 ± 0.04, 135 tubules, *n = 9* cells*, N* = 3 sheep; *p* = 0.03; figure 3*b*), indicating that possibly they penetrated more than half the cell width. The comparison of t-tubule length normalized to the cell width in the remote and border regions (remote: 0.49 ± 0.03, 132 t-tubules, *n* = 9 cells, *N = 3* sheep) revealed no difference (*p* = 0.37). Despite the lack of significance, the t-tubule length as a fraction of cell width was numerically greater in the remote cells than the control cells (*p* = 0.2). This suggests that increased t-tubule length is a remodelling feature restricted to the MI border region, although this may also have been detected in the remote region had a larger sample size been used.

#### (iv) Organized tubular network in the remote region

**Figure 5.**
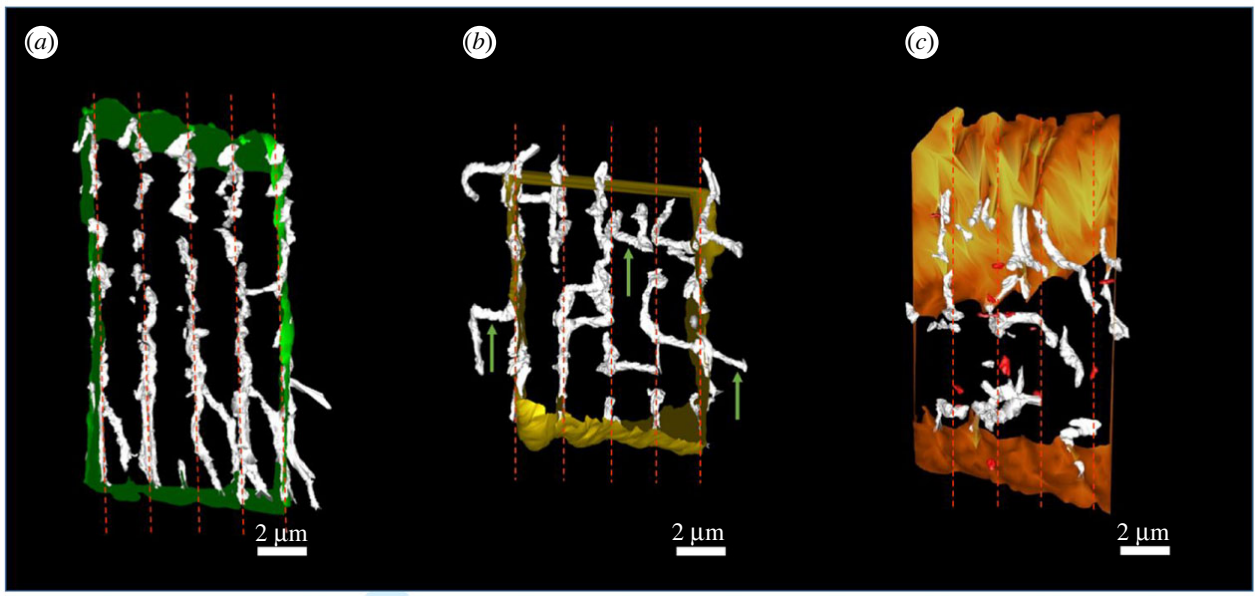
Regional involution of the tubular system from control to remote to border region MI region. (*a*) Control t-tubules (white) are regularly aligned along the *z*-lines (red dashed lines). (*b*) Remote region t-tubules (white) are aligned along the *z*-lines (red dashed lines) and present some longitudinal elements (green arrows). (*c*) Border region t-tubules (white) are irregularly aligned along the *z*-lines (red dashed lines) and are sparse and fragmented. (*a*–*c*) Scale bars: 2 µm.

3D reconstruction revealed that, similar to healthy cardiac myocytes (figure 5*a*), the tubular network in the remote region displayed an organized, regular arrangement of t-tubules along *z*-lines, with some longitudinal elements where t-tubules cross over to adjacent *z*-lines (figure 5*b*), in contrast with the highly disorganized tubular network of the border region (figure 5*c*). The volume occupied by t-tubules as a percentage of the total cell volume showed no difference between the remote region and control cardiac myocytes (remote: 1.5 ± 0.2%, 240 t-tubules, *n* = 9 cells, *N =* 3 sheep; control: 1.8 ± 0.2%, 308 t-tubules, *n* = 9 cells, *N =* 3 sheep; *p* = 0.49; figure 3*d*). t-Tubule fragmentation of the remote region was higher than in control (remote: 0.01 ± 0.004 fragments μm^−^^3^, *n* = 9 cells, *N =* 3 sheep; control: 0.0017 ± 0.001 fragments μm^−^^3^, *n* = 9 cells, *N =* 3 sheep; figure 3*f*). Interestingly, more t-tubule fragments were also observed in the remote region although the volume occupied by fragments as a percentage of the cell volume was not significantly different between remote region and control (figure 3*h*), possibly representing the very early stages of an adaptive remodelling. Extensive data on fragment volumes can be found in supplementary material.

Furthermore, analysis of t-tubule diameters showed no difference in mean diameters in the remote region and control (remote: 449 ± 13 nm, 132 t-tubules, *n* = 9 cells, *N = 3* sheep; control: 405 ± 11 nm, 135 t-tubules, *n* = 9 cells, *N = 3* sheep; *p* = 0.18; figure 3*a*). It is worth noting that t-tubule diameters were also less in the remote region compared with the border region (border: 533 ± 30 nm; figure 3*a*), indicating that t-tubular dilation did not affect the remote region. Comparison of the mean t-tubular surface area showed a decrease in t-tubule surface area in the remote region compared with the border region (remote: 4.9 ± 0.3 µm^2^, 240 t-tubules, *n* = 9 cells, *N =* 3 sheep; border: 6.8 ± 0.4 µm^2^, 227 t-tubules, *n* = 9 cells, *N =* 3 sheep; *p* = 0.01). This confirms that t-tubules of the remote region were thinner than t-tubules in the border region, in line with t-tubule diameter analysis.

## 4. Discussion

In this study, we used a translationally relevant sheep IR MI model, which was verified on a macroscale by a reduced LVEF and confirmed on a nanoscale by SBF-SEM of cardiac myocytes from regions proximal and distal to the MI focus, with the ultimate purpose to understand the remodelling occurring in the human heart during MI.

The main findings from this study were fourfold: (i) regional heterogeneity in t-tubule remodelling, (ii) fragmentation of the border region tubular network, (iii) increased width of t-tubules in the border region, and (iv) t-tubules lengthening in the border region. Together these changes likely impact on dyadic function and could contribute to regional impairment of contractility in the infarcted myocardium.

### (a) Potential clinical significance of ischaemia reperfusion model

While rodent models are cost-effective, they are of limited translational value from several aspects, including cardiac size, heart rate and dependence on cellular sources and sinks of Ca^2+^ for EC coupling [29–31]. Additionally, the sheep heart lacks a collateral coronary supply and has gross morphology similar to human [32]. Critically in the present model, coronary occlusion is transient in nature and therefore mimics more closely the human paradigm of ischaemia and reperfusion than permanent ligation models.

### (b) Organization and morphology of t-tubules in healthy sheep

In healthy sheep, the t-tubules are highly organized and arranged regularly along *z*-lines. In transverse section, t-tubules demonstrated a centripetal spoke-like arrangement, in agreement with previous work on human [3] and sheep [33] cardiac myocytes. Previous studies have reported longitudinal tubules in control cardiac myocytes [3,4], which were also observed in our nanoscale 3D reconstructions, where t-tubules cross over to adjacent *z*-lines; however, it was clear that in general, t-tubules travel in the transverse direction. As reported in previous sheep [33] and pig [34] studies, twin t-tubules were found also in this study and they may play a role in efficient action potential propagation through the cardiac myocyte facilitating EC coupling.

Our data showed that the average t-tubule diameter is similar to diameters previously reported in human and rabbit ventricular myocytes [4,35] but wider than diameters previously found in sheep [33]. This is possibly due to the fact that, we calculated the *equivalent diameter* of a t-tubule based on the assumption that all the contours segmented, to represent the cross-sections of t-tubules, are circles. This is a useful approximation which simplifies diameter calculations although it is affected by an overestimation in the case of slanted t-tubules contours.

T-tubules lengths were normalized to cell width to account for varying cell widths. We found that in control cells t-tubules penetrate about 40% of the cell thickness leaving a small portion (approx. 4% of the cell cross-section) of the cell centre unreached. In accordance with previous research in sheep [33] and similar to the data reviewed by Bers [36] for mice, guinea pigs and rats the tubular network in control myocytes appeared to occupy approximately 1.8% of the cardiac myocyte volume.

### (c) Remodelling of the tubular network in the infarct border region post-myocardial infarction

In comparison to control, t-tubules in the MI border region were not regularly arranged along the *z*-lines appearing disorganized, fragmented, sparse and dilated. Disorganization of t-tubules in cardiac failure was similarly observed in numerous studies in a variety of other species including humans [11], rats [11,37,38], pigs [17,34] and mice [2,39,40]. This disorganization could be a consequence of post-infarction collagen deposition during cardiac remodelling. For instance, Crossman *et al*. [41] reported type VI collagen in the t-tubule lumen in failing hearts, where it was associated with sarcolemmal displacement. A similar phenomenon could be responsible for the disorganization of the tubular network observed in the IR MI sheep model with subsequent effects on Ca^2+^ propagation.

In this model, we also noted extreme t-tubules disorganization in the form of t-tubules fragmentation in the border region. Vacuoles were identified in a rabbit MI model [42] and considered to be dilated remodelled t-tubules while in a sheep tachypacing heart failure model they were hypothesized to be sites of mitochondrial rupture or loss [33]. Consistent with previous studies [43] characterizing the sarcolemma basement membrane, we noted that t-tubules imaged by SBF-SEM were lined by an inner dark continuous line representing the basement membrane (figure 4*e*). This feature was used to distinguish between vacuoles formed by potentially enlarged SR or burst mitochondria and ‘proper’ fragments which are the remnants of t-tubules. In fact, all fragments were characterized by an inner basement membrane unequivocally proving their t-tubule origin (figure 4*f*). IR induces osmotic imbalance which may contribute to the fragmentation of t-tubules as it could induce changes in the expression of key genes associated with t-tubule formation and maintenance including Junctophilin 2 (JPH2), Caveolin 3 (CAV3) and Bridging Integrator 1 (BIN1) [19,44,45]. Sustained t-tubule fragmentation and recycling could also be a factor leading to t-tubule depletion.

In the border region, patchy depletion of t-tubules was also noted. This is consistent with previous studies on t-tubules loss in cardiac failure [11,34,46,47]. Surprisingly, Ohler *et al.* [13] found no changes in the tubular network in human cardiomyopathy. However, this is possibly due to selection of ‘healthy t-tubules’ during cardiac myocyte sampling, as Crossman *et al*. [3] identified that a diseased t-tubule area can coexist with a region of healthy t-tubules within the same cardiac myocyte. In the border region, longitudinal tubules were found, in accordance with findings reported in previous studies [18,48,49], but they were not as dominant a feature as fragmentation and depletion. A potential mechanism driving such changes in the border region is the enhanced wall stress experienced in this region of the infarcted myocardium which affects JPH2 downregulation disrupting the tubular structure as reported by Frisk *et al.* [46].

t-Tubules were dilated in the MI border region, in comparison to control. t-Tubules dilation is considered a common remodelling feature in cardiac failure in different species including humans [3,14], rats [7] and pigs [34]. Seidel *et al.* [14] showed that some t-tubules are dilated and compressed having a typical sheet-like appearance as opposed to characteristic column-shaped t-tubules. On the other hand, Crossman *et al.* [3] found that, while t-tubules were dilated in cardiac failure, the variance of t-tubule diameters was also increased, suggesting that not all t-tubules are dilated. In agreement with t-tubule dilation, we found that the mean surface area of t-tubules trended toward an increase in the border region, compared with control. A study on pig t-tubule remodelling [34] similarly found an increase of the mean t-tubules surface area in the peri-infarct zone following MI, confirming the dilative remodelling of t-tubules. During the late stages of cardiac remodelling following MI, myocytes become hypertrophic possibly stretching and further dilating the t-tubules. Supporting this idea, Seidel *et al.* [47] found that local strain secondary to fibrosis was associated with dilated, sheet-like remodelling of t-tubules in infarcted rabbit cardiac myocytes. Accordingly, Crossman *et al*. [41] observed the presence of collagen in the lumen of t-tubules in failing human hearts, which was associated with increased t-tubule diameters.

The total relative volume occupied by t-tubules in the IR border region did not differ significantly from control, albeit the numerical density was significantly decreased. This is, possibly, an indication that surviving t-tubules are dilating to increase the coupling with the SR and to deliver as much Ca^2+^ to the myocyte as possible to provide for the missing t-tubules and facilitate cardiac myocyte contraction.

### (d) Regional variation of t-tubule remodelling: border versus remote regions

Previous research has shown that t-tubule remodelling in cardiac failure occurs in the border zone as well as in the remote zone in rodent [17] and pig hearts following MI [50], and in the septal region of the myocardium following a large infarction of the left ventricle [2]. In addition, a study on human dilated cardiomyopathy reported that there are regional differences in t-tubule remodelling [51]. Remodelling of the tubular network in the remote region in this MI model was minimal compared with the border region with a centripetal orientation of the t-tubules located at the *z*-lines and unremarkable fragmentation or depletion. Similarly Chen *et al.* [52] identified little remodelling in the remote zone, in contrast with the striking remodelling of t-tubules in the border zone in a mouse MI model. However, in order to gain a holistic understanding of the functional consequences, it is important to consider possible alterations to the morphology of individual t-tubules in the remote region. There was no difference in t-tubule diameter between control and the remote region in our model. By contrast, Pinali *et al*. [34] injected embolization microspheres via a catheter in the LAD artery of a pig heart to create micro-infarctions after the injection point and found that t-tubules were narrower in the remote region compared with control, and suggested that this may reflect the initial stages of t-tubule remodelling. We also found that, unlike the border region, the fractional length of the t-tubules in the remote region was no different to control. While a previous study found an increase in the total length of the tubular network in cardiac failure [16] to our knowledge, we report for the first time, the increased length of individual t-tubules in the border region following MI. This could be the result of the bent and convoluted path of the t-tubules, as opposed to an increase in their radial length across the cell. Increase in t-tubule length may be a compensatory mechanism which enables longer surviving t-tubules to propagate action potentials across a larger volume of the cell maintaining EC coupling to some extent.

### (e) Transverse tubule remodelling and calcium handling

The remodelling of the tubular network has consequences for calcium handling and contractility of the heart. For example, in a three-week congestive heart failure mouse model, Louch *et al.* [2] reported that disorganization of t-tubules and irregular gaps between t-tubules resulted in slowed Ca^2+^ diffusion leading to areas of delayed Ca^2+^ release which played a role in broadening of the Ca^2+^ peak resulting in dyssynchronous Ca^2+^ release. Similarly, disorganization of the tubular network, observed here in the border region, is likely to result in disruptions to EC coupling and Ca^2+^ release dyssynchrony contributing to the development of contractile dysfunction post-MI.

Tubular detachment and fragmentation prevents propagation of the action potential through the cell volume resulting in loss of CICR and dyssynchronous Ca^2+^ release. Although LTCCs on a fragment and the RyRs on the SR may still constitute a dyad, due to the lack of connection to the sarcolemma, the LTCCs will be uncoupled from the action potential, and therefore non-functional. Additionally, any apposed RyRs will also be functionally orphaned, which will further dampen CICR. This derangement may provide a mechanism for cardiac arrhythmias by increasing the occurrence of spontaneous Ca^2+^ sparks and thence triggered activity [20].

We suggest that where t-tubules are depleted, the propagation of the action potential will begin at the sarcolemma, but will not reach the cell centre efficiently. Slowed propagation of the action potential will result in areas of delayed Ca^2+^ release and reduced CICR [2,17,19] leading to Ca^2+^ release dyssynchrony. Additionally, delayed Ca^2+^ release has been linked with a prolonged action potential duration [16], which manifests in slowed contraction [11] and contractile dysfunction. In agreement with this idea, Crossman *et al.* [51] identified that the loss of t-tubules was associated with poor contraction in human dilated cardiomyopathy.

Dilation of t-tubules may contribute to spatial displacement of the cardiac dyad, leading to LTCC-RyR uncoupling. Dyadic displacement will result in disruptions to CICR and asynchronous Ca^2+^ release. Accordingly, Crossman *et al.* [53] reported that the displacement of the dyad by 10–20 nm, secondary to t-tubules dilation could result in disruptions to SR Ca^2+^ release. However, whether such dyadic cleft widening would lead to a maintained effect on systolic Ca^2+^ release has been debated [54].

While the gross remodelling of the t-tubules in the border region has mostly deleterious effects on Ca^2+^ handling, longer t-tubules in this region could indicate an initial remodelling process which may have facilitated the propagation of action potentials in areas depleted of LTCCs within the border region, subsequently forming more LTCC-RyR dyads providing temporary compensatory routes for EC coupling in disease. In support of this, using a mouse MI model, Mørk *et al.* [55] found that the magnitude of Ca^2+^ transients was higher in infarcted cardiac myocytes, secondary to increased Ca^2+^ influx via LTCCs, which could be a consequence of increased dyad formation as proposed here, therefore, highlighting a potential compensatory adaptation of longer t-tubules post-MI.

## 5. Conclusion

We have developed a translationally relevant sheep ischaemia reperfusion MI model to reproduce as closely as possible, the remodelling changes to the tubular network which occur following MI in humans. Regional heterogeneity of the t-tubules was a characterizing feature of this model, where t-tubule depletion, dilatation and elongation were noted to occur preferentially in the border region.

Regional differences in the tubular network of the heart adapting to the ischaemic insult, highlight the necessity to develop regional therapies targeting t-tubule repair and maintenance to counteract Ca^2+^ dyssynchrony and contractile dysfunction.

## Data Availability

Summary data are available as electronic supplementary material [56].
